# Gastric metastasis from breast cancer presenting as dysphagia

**DOI:** 10.1093/jscr/rjac080

**Published:** 2022-03-17

**Authors:** Fotios S Fousekis, Kostas Tepelenis, Stefanos K Stefanou, Christos K Stefanou, George Pappas-Gogos, Vasileios Theopistos, Zoi Evangelou, Davide Mauri, Dimitrios K Christodoulou

**Affiliations:** Department of Gastroenterology and Hepatology, University Hospital of Ioannina, Ioannina, Greece; Department of Surgery, University Hospital of Ioannina, Ioannina, Greece; Department of Surgery, General Hospital of Ioannina “G. Xatzikosta”, Ioannina, Greece; Department of Surgery, General Hospital of Filiates, Filiates, Greece; Department of Surgery, University Hospital of Ioannina, Ioannina, Greece; Department of Gastroenterology and Hepatology, University Hospital of Ioannina, Ioannina, Greece; Department of Pathology, University Hospital of Ioannina, Ioannina, Greece; Department of Oncology, University Hospital of Ioannina, Ioannina, Greece; Department of Gastroenterology and Hepatology, University Hospital of Ioannina, Ioannina, Greece

## Abstract

Gastric metastasis from breast cancer occurs infrequently and causes non-specific symptoms, usually attributed to the underlying disease. Furthermore, endoscopic findings are almost identical to primary gastric cancer, making the immunohistochemical examination of biopsies necessary for diagnosis. We present the case of a 64-year-old woman who was diagnosed with lobular breast cancer 3 years ago and received chemotherapy with evidence of remission. The patient presented with dyspepsia and progressive dysphagia for the last 6 months, not responsive to PPI treatment. Upper endoscopy revealed partial occlusion of the cardio-esophageal junction and thickened gastric folds resembling linitis plastica. However, immunohistochemical analysis of endoscopic biopsies showed infiltration of gastric mucosa by lobular breast cancer cells, making the diagnosis of gastric metastasis. Therefore, clinicians’ awareness of possible gastric metastasis is warranted in patients with a history of advanced breast cancer and severe gastric symptoms.

## INTRODUCTION

Breast cancer remains the most frequently diagnosed malignancy among females and is the second most common cause of cancer death in women. Breast cancer may be classified by its anatomical origin (lobular or ductal), hormone receptivity and histological characteristics [1]. The most common metastatic sites include lung, bone, liver and brain, whereas metastases to the gastrointestinal tract from breast cancer seem to occur infrequently. The estimated rate of metastases to the stomach ranges from 0.3% in retrospective studies to 8–18% in autopsy series [2]. The differentiation between primary gastric cancer and gastric metastasis is based on clinical, endoscopic and histological findings.

In this case, we describe a patient diagnosed with breast cancer 3 years ago, who presented with dyspeptic symptoms and dysphagia and gastric metastasis. With this case report, we hope to increase clinicians’ awareness of potential stomach metastasis in patients with advanced breast cancer and severe gastrointestinal symptoms.

## CASE REPORT

The oncologist referred a 64-year-old woman to the gastroenterology clinic due to progressive dysphagia in solid food and dyspeptic symptoms starting 6 months ago. She had received a proton pump inhibitor without remission of symptoms. Her medical history consisted of metastatic lobular breast cancer from 3 years with pulmonary and bone metastases. Her breast cancer was Grade 2 of invasive lobular type with the expression of progesterone receptor (PR), estrogen receptor (ER) and lack of human epidermal growth factor receptor. She had received letrozole and palbociclib. In the last staging 4 months ago, computed tomography of thorax and abdomen showed remission of cancer and decreased tumor markers without detecting any gastric abnormalities. Physical examination revealed only mild upper abdominal tenderness.

**Figure 1:**
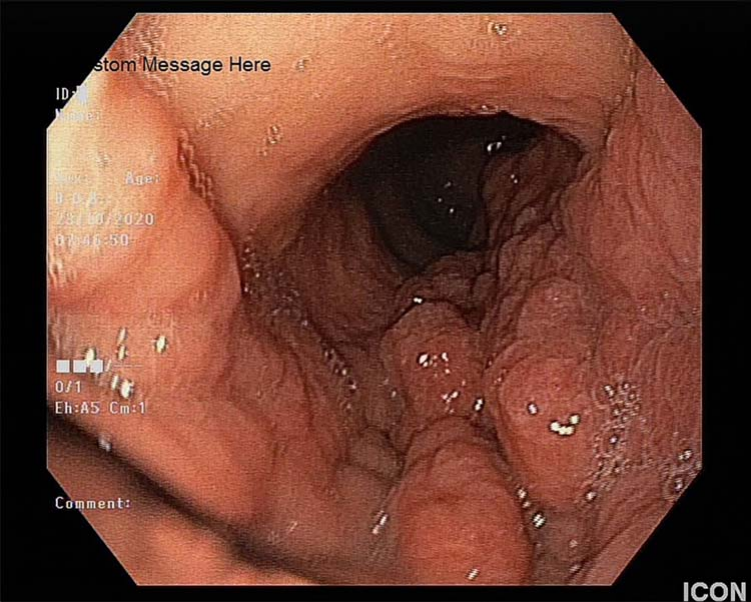
The upper endoscopy shows the thickened gastric folds and the nodular and edematous mucosa.

The patient underwent an esophagogastroduodenoscopy, which revealed an extensive neoplastic stomach lesion, which caused partial obstruction in the esophagogastric junction. The lesion involved the body, the major curvature and the stomach’s antrum. The gastric were thickened, whereas the mucosa was nodular, edematous and fragile (Fig. 1). Furthermore, a second lesion coexisted in the bulb of the duodenum. Multiple biopsies were taken.

**Figure 2:**
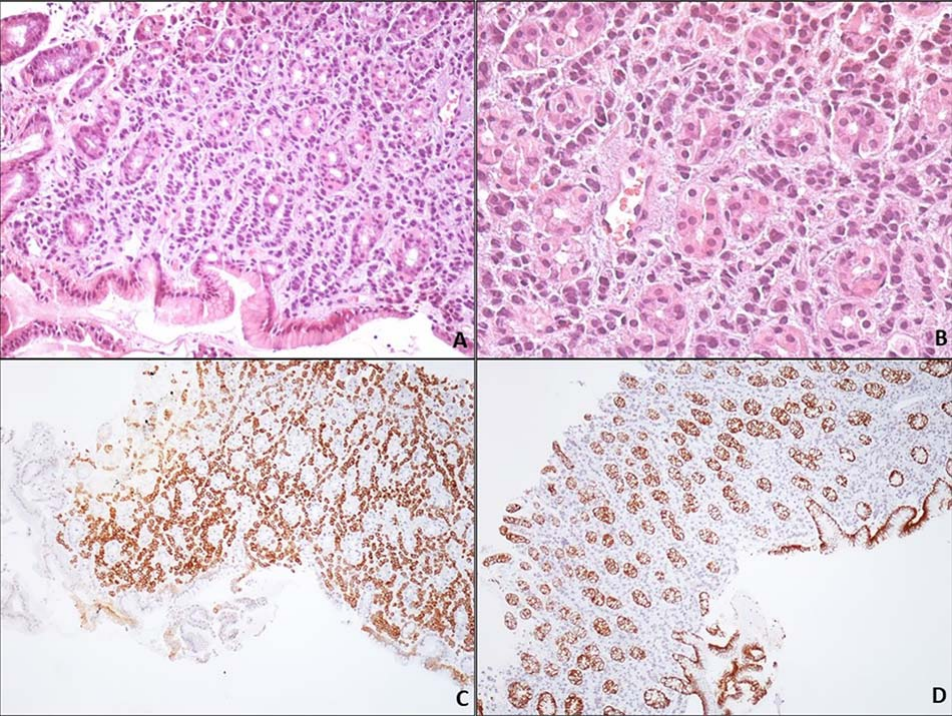
Gastric metastasis of lobular breast carcinoma. Gastric biopsy of the corpus region reveals a carcinoma arranged loosely in a linear pattern throughout the stroma between the gastric glands (**A**, H/EX200). The neoplastic cells are small, uniform, round with minimal pleomorphism; the nucleus has evenly dispersed chromatin and no nucleoli (**B**, H/EX400). Immunohistochemically, the neoplastic cells are positive for keratin 7 (**C**, H/EX10) and negative for E-cadherin (**D**, H/EX10).

Synchronous primary gastric cancer was suspected based on the endoscopic findings; however, histological examinations showed gastric infiltration by lobular breast cancer. The immunohistochemical analysis revealed that neoplastic cells were positive for CK7, Pancytokeratin, ER and PR markers and negative for E-cadherin (Fig. 2). Consequently, a diagnosis of extensive gastric metastasis was made. A nasojejunal feeding tube was placed, and the patient was considered for further chemotherapy.

## DISCUSSION

Breast cancer seems to be the most frequent metastatic cancer in the stomach (27.9%), followed by lung (23.8%), esophageal (19.1%), renal cell cancer (7.6%) and malignant melanoma (7%) and is usually diagnosed many years after the initial diagnosis and treatment. Furthermore, luminal-type or invasive lobular carcinoma tends to metastasize more frequently to the stomach than other breast cancer types [3].

The phenomenon of metastatic dormancy could explain the latency period of gastric metastasis of breast cancer. The disseminated tumor cells present disruption of crosstalk between growth factor and adhesion signaling, which leads to the inability of tumor cells to recruit blood vessels despite active proliferation, preventing residual tumor cell expansion [4]. In addition, the dormant disease seems to be chemotherapy-resistant because chemotherapeutic agents act on proliferative cells. Genetic and epigenetic changes, microangiogenesis and changes in the immune system and microenvironment may contribute to the regrowth of cancer cells. Due to the dormancy of gastric metastasis, some authors have proposed upper endoscopy screening in patients with breast cancer and mainly invasive lobular cancer [5].

The clinical manifestations of gastric metastases are non-specific. They are usually attributed to the location and the underlying disease. The patients may suffer from nausea, abdominal pain, bleeding, weight loss, anorexia, dyspepsia and early satiety, whereas cases of gastric perforation secondary to gastric metastasis from breast cancer have been reported [6]. In our case, the patient presented with dyspepsia and dysphagia.

Endoscopically, the most common finding of gastric metastasis is stiffness of the stomach due to diffuse infiltration, such as in linitis plastica, whereas ulcerated nodular invasion and external invasion are less frequent types of gastric metastasis. Biopsies may be normal in ~50% of patients because they are in many cases superficial, whereas breast cancer infiltration is often limited to the submucosal and seromuscular layers. Consequently, extensive and deep biopsies are mandatory for diagnosis [7, 8].

In histological examinations, signet ring cells may be demonstrated, making the differentiation difficult between primary gastric adenocarcinoma and metastatic breast cancer. Immunohistochemical markers play a crucial role in final diagnosis as these lesions may endoscopically and histologically mimic gastric cancer. Also, it is worth mentioning that invasive lobular breast cancer metastases usually keep the same expression of receptors as the primary breast cancer. The ER expression and loss of E-Cadherin should be considered diagnostic for gastric metastasis from breast cancer [9], as in our case.

The treatment of breast metastasis to the stomach includes chemotherapy and hormonal agents, whereas the role of surgical treatment, such as gastric resection and endoscopic therapy, such as endoluminal stents for gastric outlet obstruction, are limited and should be considered in palliative care for the relief of obstructive symptoms [5]. The median survival of patients diagnosed with gastric metastases is ~11 months [10].

In conclusion, metastatic involvement of the stomach secondary to breast cancer presents a long latency phase disease and manifests with non-specific symptoms, whereas immunohistochemistry is essential for diagnosis. Consequently, gastric metastasis may be held accountable for any gastrointestinal symptom in patients with advanced breast cancer.

## AUTHORS’ CONTRIBUTION

FFS and TK conceptualized and designed the study; SSK and SCK were involved in literature search and acquisition of data; P-GG and TV were involved in analysis and interpretation of data; EZ led the drafting of the manuscript; MD did critical revision; CDK approved the final version and has responsibility for its final content. All the authors agreed to be accountable for all aspects of the work in ensuring that questions related to the accuracy or integrity of any part of the work are appropriately investigated and resolved.

## CONFLICT OF INTEREST STATEMENT

None declared.

## FUNDING

None.

## CONSENT

Written informed consent was obtained from the patient for publication of this case report and accompanying images. A copy of the written consent is available for review by the Editor-in-Chief of this journal on request.
